# Editorialets

**Published:** 1911-04

**Authors:** 


### Editorialets.

Dr. Fred J. Combe, San Antonio, President Equitable Life Insurance Company, and formerly mayor of Brownsville, was married March 21st to Mrs. Carrie Moore Fordtran, of San Antonio.

The headquarters of the Pure Food and Drug Commissioner, Dr. J. S. Abbott, by act of the Legislature, will be removed from Denton to Austin, under the ægis of the State Health Department.

I have been with the "Red Back" since "Heck was a pup," and will be with you to the end.

Your friend,
A. H. McCord,
Rusk, Texas.

Dr. Harrower's new journal, *Physiologic Therapeutics,* is, indeed, "a thing of beauty"—and a mine of interesting reading. The March number is a symposium on "hypertension." Dr. Harrower will send a copy on request, and I advise my readers to subscribe for it,—a valuable addition to your list. Such journals are rare, and not easily obtained.

Another Martyr to Science.—Dr. E. F. Ainsley, the bacteriologist of the New York State Board of Health, who looked after the health of immigrants, became infected with cerebrospinal meningitis while handling certain Greek immigrants suffering with that dread disease, lost his life, despite Dr. Flexner's treatment with the antitoxin discovered and introduced by Dr. Flexner.

William W. Mayo, M. D., of Rochester, Minn.—Dr. Mayo, the father of the two celebrated Rochester surgeons of the present time, died on Monday, March 6, in the ninety-second year of his age. He was a native of England, but came to this country at an early age. He went to Rochester in 1863, having practiced previously in La Porte, Ind., and Leseuer, Minn. He retired from practice several years ago.

I have dropped fifty-three more delinquents—deadbeats—and have kept a list of them. Some of them are high in the councils. I have sometimes thought of publishing the list as a surprise to my readers. When interrogated by insurance companies—as I am, sometimes—as to the character of certain Texas doctors, I "have a little list" to which I refer, and write according to what I find there. I shall not be at Amarillo. Please remit.

---

I DON'T KNOW just how long I have been a subscriber to the "Red Back," but I do know that so long as I practice medicine, whether in Oklahoma, Texas or elsewhere, I want to keep the JOURNAL *coming*. May its editor live long and prosper.

Yours fraternally,

T. J. DODSON,

Mangum, Okla.

---

PREPARE FOR HYGIENE CONGRESS.—At the request of President Taft, who conveyed his request through the health officers of the several States, Governor Colquitt yesterday named Dr. C. W. Trueheart, of Galveston; Dr. John T. Moore, of Houston; Dr. S. R. Burroughs, of Buffalo; Dr. O. I. Halbert, of Waco, and Dr. A. L. Lincecum, of El Campo, as a delegation to organize the Texas end of the work of the International Congress of Hygiene and Demography and prepare a Texas exhibit for this congress.

---

CORINNA BORDEN KEEN RESEARCH FELLOWSHIP OF JEFFERSON MEDICAL COLLEGE.—The accumulated income of this fund now amounts to $1000. The fellowship will be awarded by the trustees upon recommendation of the faculty to a graduate of the Jefferson Medical College of not less than one, nor more than ten, years' standing, upon condition that he shall spend at least one year in Europe, America, or elsewhere, wherever he can obtain the best facilities for research in the line of work he shall select, after consultation with the faculty; and that he shall publish at least one paper embodying the results of his work as the "Corinna Borden Keen Research Fellow of the Jefferson Medical College."

Address: J. W. HOLLAND, Dean.

---

THE FOLLOWING DOCTORS from Texas are doing post-graduate work at the New Orleans Polyclinic: Drs. T. A. Moore, Greenwood; H. T. Colter, Rockdale; D. A. Mann, Diboll; Jas. L. Hill, Winnsboro; W. A. Whiteside, Timpson; S. G. Sevier, Oenaville; D. B. Beach, Quanah; S. A. Roberts, San Diego; J. D. Gray, Shiner; G. M. Stevens, Beeville; Wm. S. Irwin, Falfurrias; G. R. Barnes, Trinity; J. W. Hargrove, Como; J. V. Hopkins, Victoria; E. D. Pope, Silsbee; J. W. Gregory, Big Sandy; C. H. Brooks, Jefferson; O. D. Radney, Mt. Calm; O. L. A. Torbett, Marlin;

W. B. Cline, Midway; J. K. Smith, Texarkana; Houston Neely, Beeville.

---

THE CONSTITUTIONALITY of the one-board medical act of the Thirtieth Legislature is again attacked before the Court of Criminal Appeals. This time in the case of W. J. C. Germany vs. the State, from Tom Green county, the act is attacked as being discriminatory in that it makes no provision for practice by masseurs, although the massage has become to be recognized as a scientific treatment, and there are schools and colleges where it is taught and diplomas are given.

It is maintained that the provision of the act forbidding the practice is not properly a police regulation, and that the act is wholly unconstitutional because of this discrimination.

Germany was convicted in the county court of Tom Green county, fined $50 and given one hour in jail for practicing medicine as a physician contrary to this act without a license.

---

KRESO—A HOUSEHOLD BLESSING.—Lister became immortal by the discovery that "germs" cause disease. He was the father of bacteriology, and antiseptic surgery. His recommendation was carbolic acid. But something was needed less dangerous, less poisonous, safer, for disinfecting premises, and Parke, Davis & Co.—the benefactors—have furnished the ideal. It is Kreso. I find it most valuable for disinfecting and deodorizing, and keep it constantly on hand for use in the bathroom, the water closet, the kitchen sink, and, in fact, everywhere about the premises where germs may exist, where flies or mosquitoes may breed. It is a sweetener. Sprinkled over the manure heap, it will stop the breed of the deadly fly. Moreover, it is inexpensive. To use the trite saying, "No family should be without it." In the November 5th issue of the *American Medical Journal* there is an article highly commendatory of Kreso, but, as all my Texas subscribers are, *nolens volens* or *will-he nilhe* readers of the Octopus, I do not reproduce it here; they have all doubtless seen it.

---

BEWARE OF ITINERANT MORPHINE AND COCAINE TABLET PEDDLERS.—Manufacturers, jobbers, dispensers and others obliged to keep considerable stocks of morphine, cocaine and various forms of tablets of the same suffer considerable loss from theft, owing to an illegitimate demand and the ease with which the drugs are concealed about the person.

A man described as about 5 feet 10 inches, light brown hair, wearing a light gray overcoat, well dressed, nice looking, with a nice way, seeming to know the price of morphine and cocaine, etc., called on the Hart-Schaeffer Drug Co., Omaha, Neb., about January 23d, and tried to sell 5000 H. T. 26 P. D. & Co. at $5 per M; said he had sold 30,000 already and had 5000 more to sell. Refused to call again to see Mr. Hart. When the clerk tried to get finishing number he took package away from him with the remark: "If you don't want them o. k., I can sell them without any trouble." He claimed to be from the East—either New York or Philadelphia—saying he sold different things at different times, and now was the time to get in easy, as morphine, etc., was going up all the time.

---

For Texas Medical Journal.

THE WAR CRY OF THE BOSS.

Be loyal to the party is the war cry of the boss,
Be loyal to the party, the party, that means us.
For we're the ones to say,
And the others must obey
If they're loyal to the party, the party—that means us.

The caucus of the party is a very sacred thing,
It's decrees are law and gospel, for it's managed by the ring,*
Then we'll let the people holler,
They have got to wear the collar,
For the party they must follow, and I'm the party's king.

The insurgents are a menace to the party, don't you see,
They may represent the people, but that cuts no ice with me,
For I tell you I'm the party.
And I'll teach each little smarty
To be loyal to the party, the party—that means me.

—J. LEVERETT, M. D., Yonkers, N. Y.

---

AN ECHO FROM THE PAST.—The yellow fever in Holly Springs, Mississippi, in 1878. The illustrious Swearingen wrote the following lines with a lead pencil on the whitewashed walls of the courthouse. It has been framed and glass-covered and stands today a memento of those sad days as well as of the beloved and lost Swearingen. I reproduce it at the request of Dr. Chesley Daniel, of Holly Springs.—D.

"Within this room September, 1878, Sister Corinthia sank into

*That is, the House of Delegates.—D.

the sleep eternal. Among the first to enter this realm of death, she she was the last, save one, to leave.

"The writer of this humble notice saw her in health, gentle but strong, as she moved with noiseless step, and serene smiles through the crowded wards; he saw her when the yellow-plumed angel threw his golden shadows over the last sad scene, and eyes unused to weeping paid the tribute of tears to this brave and beautiful spirit of mercy.

"She needs no slab of Parian marble
With its white and ghastly head
To tell wanderers in the valley
The virtues of the dead.

"Let the lily be her tombstone,
And dewdrops, pure and bright,
The epitaph the angels write,
In the stillness of the night.

R. M. Swearingen, M. D.,
Austin, Texas."

---

**For Texas Medical Journal.**

### Sterilization—A Medical Ruse.

Could we sterilize the grafter, indeed,
And stop him from making seed,
If this be true, then what a ruse?
How much of this, then, will you choose?
The grafter's a fake, a quack, indeed,
Then sterilize and stop the breed.
We have a thought that's coming, too;
Then sterilize and start anew.
We've found a man, and looked him o'er;
He'll weigh two hundred pounds or more.
Why! he reaches up six feet and four,
He scarce can stand on a street car floor.
Ah! a woman we have in sight,
To mate them, then, would be just right.
She is a blonde, and American true;
She'll weigh one hundred eight-two.
Ah! such a pair, then mated right,
Would bring out all the good in sight.
Now, why delay the thing so long,
Go at it, then, and stop the throng.

I'm sixty now, me you can pass,
Of course, you know, Alas! Alas!
Now, my dear friends, don't get cross,
For to us it is no loss.
There's many a ruse being published today
There's none equals this, I'm sure you will say.
We find in reading the Good Old Book
The sterilizer to be a crook.

M. D., San Antonio.

He's played out at sixty, poor fellow, and now, like Solomon, he cries "all is vanity."—D.

